# Can Inferred Provenance and Its Visualisation Be Used to Detect Erroneous Annotation? A Case Study Using UniProtKB

**DOI:** 10.1371/journal.pone.0075541

**Published:** 2013-10-15

**Authors:** Michael J. Bell, Matthew Collison, Phillip Lord

**Affiliations:** 1 School of Computing Science, Newcastle University, Newcastle upon Tyne, United Kingdom; 2 School of Chemical Engineering and Advanced Materials, Newcastle University, Newcastle upon Tyne, United Kingdom; UGent/VIB, Belgium

## Abstract

A constant influx of new data poses a challenge in keeping the annotation in biological databases current. Most biological databases contain significant quantities of textual annotation, which often contains the richest source of knowledge. Many databases reuse existing knowledge; during the curation process annotations are often *propagated* between entries. However, this is often not made explicit. Therefore, it can be hard, potentially impossible, for a reader to identify where an annotation originated from. Within this work we attempt to identify annotation *provenance* and track its subsequent propagation. Specifically, we exploit annotation reuse within the UniProt Knowledgebase (UniProtKB), at the level of individual sentences. We describe a visualisation approach for the provenance and propagation of sentences in UniProtKB which enables a large-scale statistical analysis. Initially levels of sentence reuse within UniProtKB were analysed, showing that reuse is heavily prevalent, which enables the tracking of provenance and propagation. By analysing sentences throughout UniProtKB, a number of interesting propagation patterns were identified, covering over 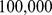 sentences. Over 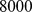 sentences remain in the database after they have been removed from the entries where they originally occurred. Analysing a subset of these sentences suggest that approximately 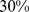 are erroneous, whilst 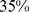 appear to be inconsistent. These results suggest that being able to visualise sentence propagation and provenance can aid in the determination of the accuracy and quality of textual annotation.

Source code and supplementary data are available from the authors website at http://homepages.cs.ncl.ac.uk/m.j.bell1/sentence_analysis/.

## Introduction

Biological databases store, organise and share ever-increasing quantities of data [1]. In addition to storing raw biological data, such as protein sequences, many databases aim to attach *textual annotation* to a given database entry. This textual annotation provides a mechanism to convey understanding of the underlying biology, providing information such as protein function and subcellular location. In describing the current knowledge about the database entry, textual annotations can form the foundations for further research [2] emphasising their crucial role in biological databases.

The quality and correctness of textual annotations inevitably varies between databases and entries. This can depend on many factors, such as: the current evidence supporting the function of the protein; the curation and review process; and the curators' judgement in extracting information from biomedical literature [3], [4]. The kind of metadata describing annotations also varies between databases and entries, limiting the ability to compare them. For example, the source (or *provenance*) and last updated date of a Gene Ontology (GO) annotation is not always apparent [5].

At the highest level, we can distinguish between two types of annotation curation process: manual curation and automated curation. It is generally held that manual curation is of higher quality and correctness than its automated counterpart. This is mainly because expert curators have the ability to access, evaluate and interpret a wide range of scientific literature as a source of information for annotations (as is the case for UniProtKB/Swiss-Prot [6] and FlyBase [7]). However, automated annotation pipelines, such as UniRule [8], provide greater annotation coverage and more regular updates, as annotations are often transferred from existing annotations.

Database sizes are continuing to expand at an exponential rate, resulting in a continued and growing reliance on automated curation. Identification of textual annotation that could be of interest in the curation process is often based upon biological sequence; sequences that share properties, such as sequence similarity, are more likely to share a similar function and attributes. Given a strong sequence similarity, it is reasonable that annotations may be copied verbatim between entries, i.e. sentences are subjected to reuse. Therefore, annotations are often based purely, or in part, on existing annotations. It is also becoming an increasingly common practice for manual curators to use existing annotations within their curation process; either from annotations within the existing database (as is the case for UniProtKB/Swiss-Prot) or from external databases (e.g. FlyBase uses UniProtKB as a source). If a database lacks formal provenance and metadata, it may mean that it is not possible to identify the original source of an annotation. Given this, the extracted textual annotation may have also previously been copied from other entries. Should the original source of a textual annotation be found to be erroneous, there is no clear way of identifying where it has propagated to.

A number of studies have explored textual annotation quality (see, for example, Bell *et al.* [9]), however, very limited work has explicitly explored textual annotation propagation and its link to correctness. One such study [10] explores the usage of association rules to detect possible erroneous annotations. This study, performed on the Swiss-Prot database, focused primarily on the annotation within the feature table; free text annotation (those within the “CC” lines) were omitted from the analysis. The reason for this omission was given as “[textual annotation] is not easily machine-parseable”. Unlike structural annotation, textual annotation is historically developed for human consumption, rather than for computational interpretation [11]. Essentially, this means textual annotations are mostly made up of free-text English.

Although textual annotation studies are limited, several explore ways to model propagation of structural annotation errors [12]–[14]. Structural annotation sits between nucleotide sequences and textual annotations; it identifies genomic elements, such as open reading frames, for a given sequence. This is similar to textual annotation, in that structural annotation often makes use of sequence data and can be manually or automatically curated. These studies [12]–[14] highlight a number of reasons for structural annotation errors, such as mis-identification of homology, omissions or typographical mistakes, concluding that annotation accuracy declines as the database size increases. Further studies attempt to actually estimate the error rates in structural annotation. These include an estimated error rate of between 28% and 30% in GOSeqLite [15] and between 33% and 43% in UniProtKB/Swiss-Prot [10]. Therefore, it is highly plausible that these errors will affect textual annotation, as acknowledged by Gilks *et al.* [12].

We hypothesise that sentence reuse is prominent within textual annotations and a lack of formal provenance has led to inaccuracies in the annotation space. Within this paper we aim: to quantify sentence reuse; to investigate patterns of reuse and provenance, through a novel visualisation technique; and to investigate whether we can use patterns of propagation to identify erroneous textual annotations, inconsistent textual annotations or textual annotations with low confidence.

## Materials and Methods

### The UniProt Knowledgebase (UniProtKB)

Analyses for this paper focus solely on annotation within UniProtKB [16]. There are a number of reasons for this. Firstly, UniProtKB consists of two sections: Swiss-Prot, which is manually curated and reviewed, and TrEMBL, which is automated and unreviewed. Secondly, the resource is well supported with an approachable helpdesk and extensive documentation, such as the UniProtKB user manual [17]. Finally, UniProt makes available all past major releases of both Swiss-Prot and TrEMBL, with the exception of Swiss-Prot versions 1–8 and 10 which were never archived, in flat file format. UniProtKB also exports the database in XML format, which includes various levels of evidence tagging [18]. However, unlike the flat file format, the XML file format is not available for all versions of the database. Further, an evidence tag shared between different pieces of data could be interpreted differently by different users, and thus account for different sentences. Therefore, the flat file format is used.

Therefore, UniProtKB provides an ideal resource to compare textual annotation reuse within manually and automatically curated resources and to investigate its propagation. Since the first version of Swiss-Prot and TrEMBL a number of key changes in the release process have occurred. Prior to the formation of the UniProt Consortium, the releases of Swiss-Prot and TrEMBL were not synchronised; TrEMBL was released more frequently than Swiss-Prot. In 2004, these releases became synchronised with UniProtKB initially distinguishing between major and minor releases until version 15, when this distinction was abandoned; UniProtKB releases are now on a four week cycle. These changes make comparison and discussion somewhat challenging, so we will use the following naming conventions for clarity:

- **UniProt** – Refers to the UniProt Consortium.
- **Swiss-Prot** – Refers to Swiss-Prot database releases prior to the formation of the UniProt Consortium.
- **TrEMBL** – Refers to TrEMBL database releases prior to the formation of the UniProt Consortium.
- **UniProtKB** – Refers to the UniProt Knowledgebase, including both Swiss-Prot and TrEMBL databases.

Where necessary we will explicitly write UniProtKB/Swiss-Prot or UniProtKB/TrEMBL. This naming scheme allows us to refer to post-UniProtKB versions of UniProtKB/Swiss-Prot and UniProtKB/TrEMBL with the same number, starting from version two of UniProtKB, which was the first major release, containing Swiss-Prot version 44 and TrEMBL version 27. This numbering scheme continues until version 15, when subsequent versions follow the format YYYY_MM, starting from 2010_01. Complete datasets for historical versions of UniProtKB and Swiss-Prot are made available by UniProt on their FTP server (ftp.uniprot.org/pub/databases/uniprot/). Pre-UniProtKB/TrEMBL releases were kindly made available to us by UniProt.

### Sentence extraction

Our extraction process has two key parts: a custom made parsing framework to extract and format the comment lines from UniProtKB entries and a program to extract the sentences from these formatted comment lines. The correct extraction of sentences from text is not straightforward. Given this, we utilised the LingPipe tool kit [19]; a suite of Java libraries for processing text.

**Figure 1:**
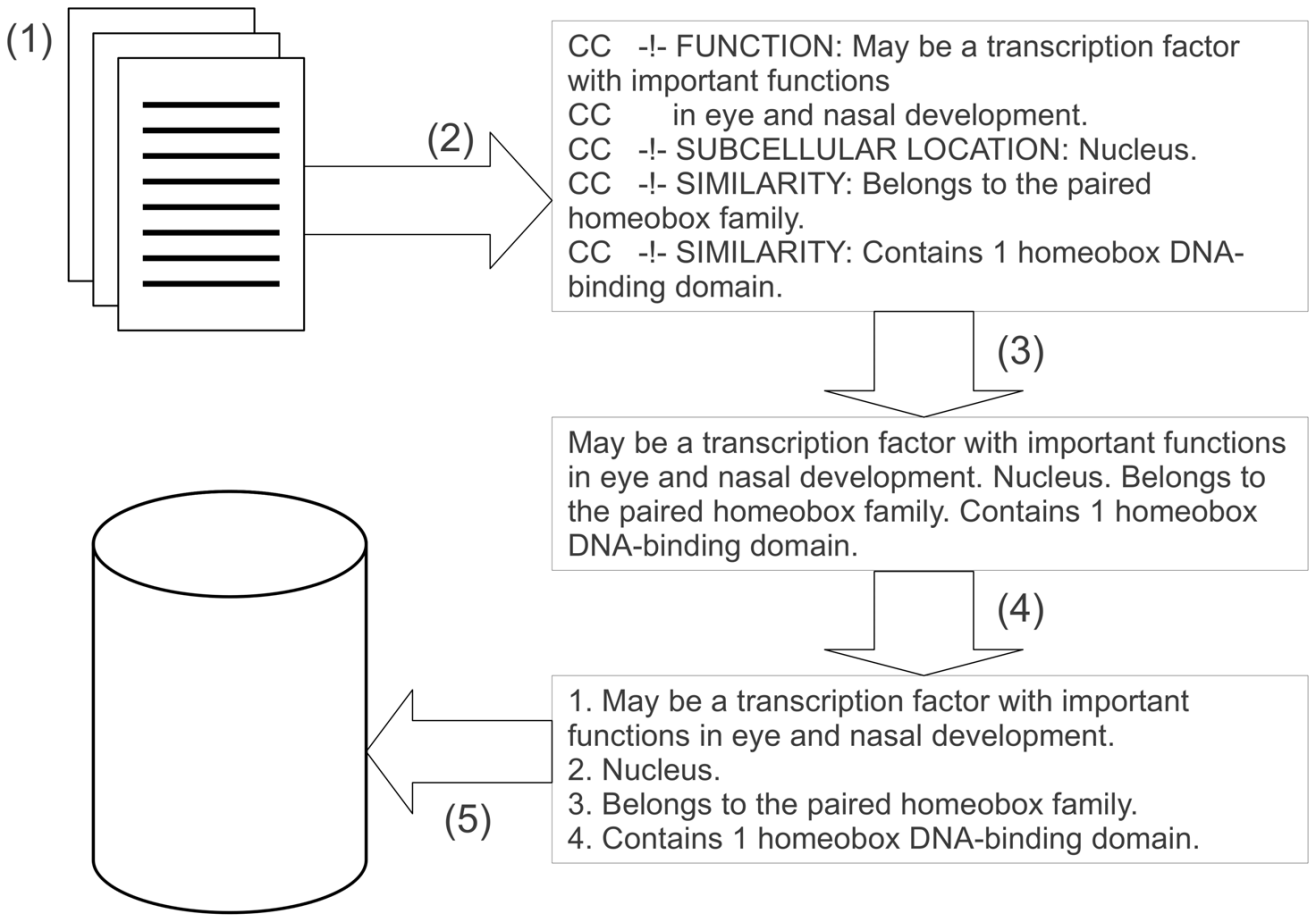
Outline view of the data extraction process. (1) Initially we download a complete dataset for a given database version in flat file format. (2) We then extract the comment lines (lines beginning with ‘CC’, the comment indicator). (3) We remove comment blocks and properties (as defined in the UniProtKB manual [17]) and the ‘CC’ identifier. (4) Sentences are then extracted, using LingPipe. (5) Finally, all of the identified sentences are added to the MySQL database.

A typical sentence within our work is one which contains a group of words and is terminated with a full stop. However, there are a number of exceptions to this basic rule, such as abbreviations, which are especially commonplace in the biomedical domain. The vast majority of these are handled correctly by LingPipe. However, the structure of textual annotation in UniProtKB can mean topic blocks or lists are not terminated with a full stop. In cases such as these, we count these as sentences. Specifically, our extraction process, as summarised in Figure 1, involves:

1. Downloading and extracting complete datasets from historical versions of UniProtKB in flat file format from the UniProt FTP server.
2. Extracting comment lines from these flat files using the Java framework created to handle the UniProtKB flat file structure, as detailed in the UniProtKB user manual [17].
3. Removing topic headings, the “CC” identifier and copyright and licence statements. Over time annotations in UniProtKB have become more structured with the addition of topic headings (e.g. “subcellular location” and “function”) in the comments lines, which were removed to maintain sentence integrity.
4. Extracting a list of all the sentences from each entries comment lines using LingPipe.
5. Storing extracted sentences in a MySQL database, stating the entry it appears in and for which database version.

To ensure that annotation data was correctly extracted from UniProtKB, a number of checks were performed. These checks mostly involved making use of the UniSave tool [20], made available by UniProt. UniSave allows the differences within an entry to be compared between two different versions. Making use of this tool we were able to manually check that sentences were correctly parsed for a random selection of entries and versions.

Once we extracted all of the sentences from every entry within a given database version, we had a set of sentences which we refer to as the *total* number of sentences within a database version. This set of sentences is redundant, as a number of sentences will occur multiple times within the set. Taking each sentence from this set only once (i.e. extract the distinct sentences) results in a set of non-redundant *unique* sentences. Finally, within a set of unique sentences, some sentences occur only a single time within a database version; that is they are *singleton* sentences. We can summarise these definitions as:

- **Total sentences** – A redundant set of all sentences in a database version.
- **Unique sentences** – A non-redundant set of all sentences in a database version.
- **Singleton sentences** – A set of sentences that occur only a single time within an entire database version.

## Results

### How heavily reused are sentences in UniProtKB?

**Figure 2:**
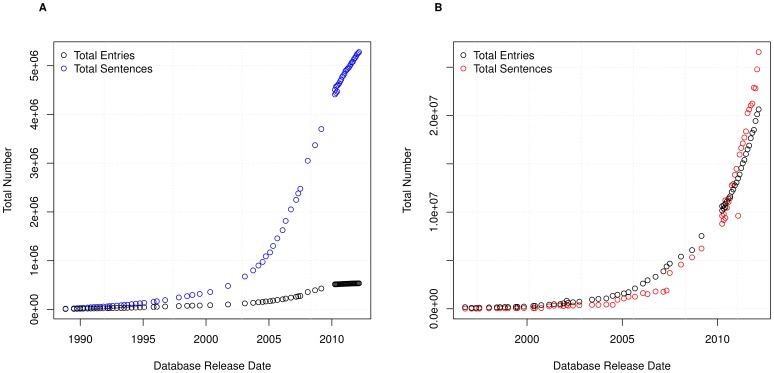
Total sentences and entries. The total number of sentences and entries in (A) Swiss-Prot and (B) TrEMBL.

The curation process implemented by UniProtKB [6] means that sentences will be reused. To understand the amount of sentence reuse over time, we initially analyse the total number of sentences that are used within each version of Swiss-Prot and TrEMBL. These results, as shown in Figure 2, clearly show that the total number of sentences is growing rapidly. Whilst the growth for Swiss-Prot shows a relatively regular progression, TrEMBL has a somewhat more irregular and disjointed growth. Figure 2 also shows the number of entries within UniProtKB over time. This figure shows that the growth of sentences within TrEMBL generally follows the growth of the database, whilst the growth of sentences in Swiss-Prot is much slower than the growth of entries.

**Figure 3:**
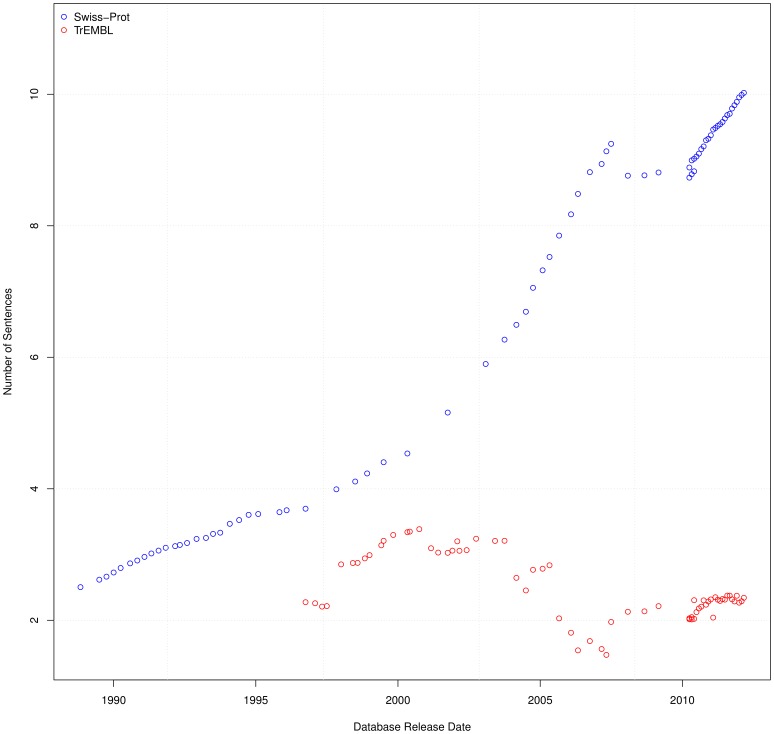
Average sentences per entry. The number of sentences that appear, on average, in each entry in TrEMBL and Swiss-Prot (i.e. the total number of sentences divided by the total number of entries).

Both the size of the database and the number of sentences is increasing over time. How, then, does this impact the way sentences are re-used and distributed within the database? We can gain an insight into the re-use by analysing the average number of sentences per entry, as shown in Figure 3. For this calculation, only entries containing textual annotation were considered. Figure 3 shows that, over time, entries within Swiss-Prot have an increasing number of sentences in their annotations. Over a twenty year period, Swiss-Prot has seen the number of sentences within the textual annotation of an entry increase fivefold, to the current average of around ten. Conversely, TrEMBL has seen fluctuations over time, but typically remained at an average of around two sentences per entry.

**Figure 4:**
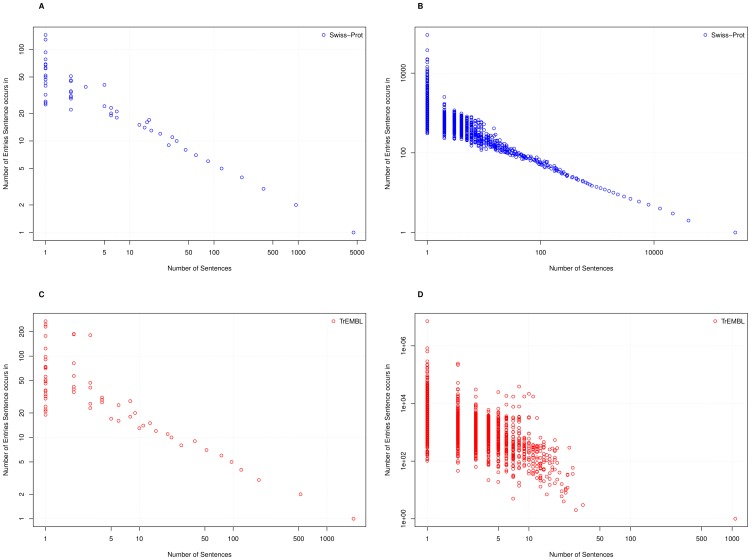
Showing the distribution of sentence reuse through Swiss-Prot and TrEMBL. (A) Swiss-Prot Version 9 (B) UniProtKB/Swiss-Prot Version 2012_05 (C) TrEMBL Version 1 (D) UniProtKB/TrEMBL Version 2012_05. As an example, in Figure A, the bottom right point states that there is ∼5000 sentences that occur a single time, whilst the top-left-most point indicates that there is one sentence that occurs ∼125 times.

To complement this reuse analysis, we can also analyse how sentences are distributed throughout UniProtKB, as shown by Figure 4. This shows the distribution is much more even in Swiss-Prot than TrEMBL, whilst again highlighting the increasing levels of reuse over time.

**Figure 5:**
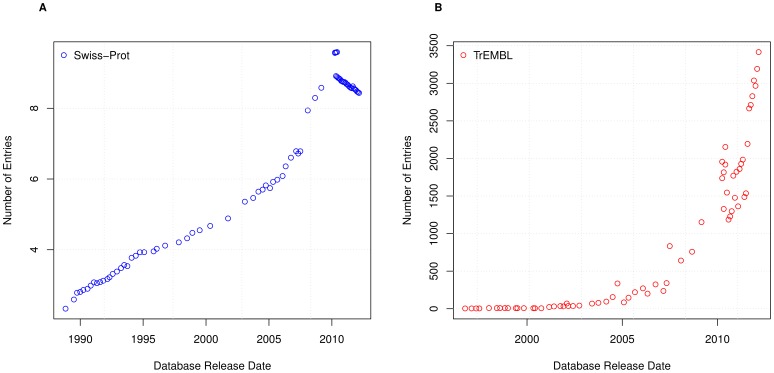
Average Entries Per Sentence. Graph showing the average number of entries that each sentence appears in for (A) Swiss-Prot and (B) TrEMBL.

The increase in the number of sentences in the textual annotation per entry over time fits with one of the goals of UniProtKB, which is to attach as much information as possible to each protein entry [21]. A significant amount of this increase is through sentence reuse. We can see this by considering the number of entries that each unique sentence occurs in; Figure 5 shows that the average number of entries where a particular sentence appears is generally increasing for Swiss-Prot and TrEMBL, to a current average of about 9 and 3500 respectively; interestingly later versions of Swiss-Prot are starting to show a decline in reuse, which coincides with the change in release cycle of UniProtKB.

These results suggest that whilst total textual annotation is increasing for entries on average, it is driven by sentence reuse. Another factor affecting the amount of sentence reuse could be UniProtKB attempting to reduce the number of entries that remain without any textual annotation.

**Figure 6:**
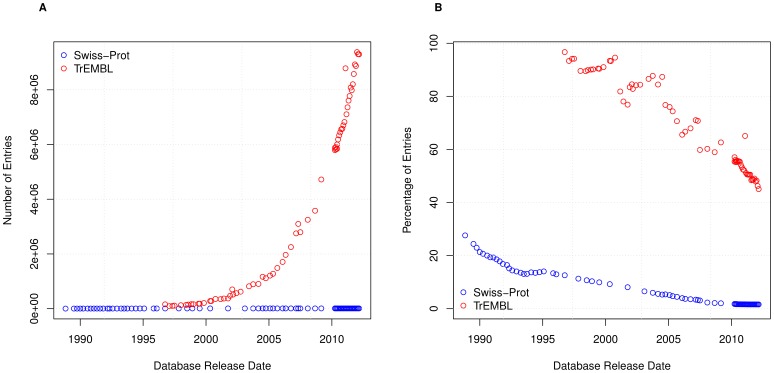
Entries without annotation. (A) Number of entries in TrEMBL and Swiss-Prot without any annotation, and (B) the percentage of entries without any annotation.

We show the number of entries without any textual annotation in Figure 6 (A), and the overall percentage in Figure 6 (B). Over time the overall percentage of these entries is decreasing; only around 1.5% of entries in the latest version of Swiss-Prot contain no textual annotation, compared to 45% of entries in the latest version of TrEMBL. Both of these show significant improvements over time – initially Swiss-Prot had 27.6% of entries without any textual annotation in 1988 compared to TrEMBL which had 96.7% in 1996.

Therefore, we conclude that, in addition to the increase of the overall database size, the percentage of entries with annotation is increasing; these two factors both contribute to the increasing reuse of sentences.

These results suggest that the amount of textual annotation is increasing due to an increase in sentence reuse. We therefore want to abstract from the overall reuse and ask: how is the number of unique sentences changing over time?

**Figure 7:**
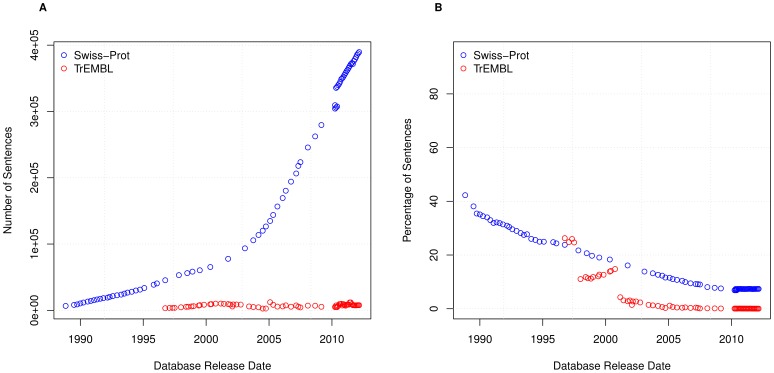
Unique sentences. (A) The number of unique sentences in Swiss-Prot and TrEMBL and (B) the percentage of unique sentences in Swiss-Prot and TrEMBL.

Figure 7 (A) shows the level of unique sentences within both TrEMBL and Swiss-Prot. From this, it is immediately clear that sentences are much more heavily reused within TrEMBL than Swiss-Prot. To further illustrate this, in Figure 7 (B) we show the percentage of unique sentences for each database version of Swiss-Prot and TrEMBL. This graph shows a steady decline in both Swiss-Prot and TrEMBL, providing further evidence that sentence reuse in both databases is on the rise. For example, within UniProtKB/TrEMBL Version 2012_05 there are approximately 22 million entries, containing approximately 26.7 million sentences, 8131 of which are unique; i.e. the entire TrEMBL sentence corpus is made up of only 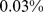 sentences.

**Figure 8:**
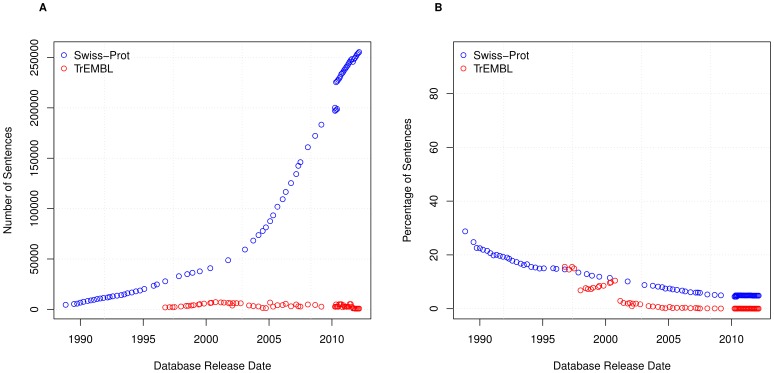
Singleton sentences. The number of singleton sentences in Swiss-Prot and TrEMBL and (B) the percentage of singleton sentences in Swiss-Prot and TrEMBL.

A special case of the unique sentence is the singleton sentence, that is a sentence which occurs once, and only once, within a database version. The number of singleton sentences is shown in Figure 8 (A) with the percentage shown in Figure 8 (B). Figures 8 and 7 show both singleton and unique sentences follow an almost identical pattern.

In all cases we see that while absolute numbers are increasing, percentages are decreasing. We see this for both unique sentences (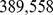 and 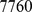 (∼7% and ∼0.03%) in the latest versions of Swiss-Prot and TrEMBL, respectively) and singleton sentences (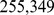 and 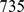 (∼5% and ∼0.003%) in the latest versions of Swiss-Prot and TrEMBL, respectively). Therefore, in conclusion, this shows that reuse between records is increasing in both Swiss-Prot and TrEMBL, and this trend appears set to continue.

Whilst we are able to quantify this reuse, we are currently unable to analyse and depict the reuse of individual sentences; we would like to analyse and explore how an individual sentence is propagated through the database. For this analysis to be performed we need to identify possible visualisation approaches, as explored in the following section.

### How can we visualise sentence propagation?

We have shown that sentence reuse in UniProtKB is both common and increasing. Therefore, given the scale of this data, we explore the usage of *visualisation*. By visualising sentence reuse across entries and over time, we may be able to better understand annotation propagation and infer provenance. From this, it is possible that patterns demonstrating interesting traits in the underlying data may emerge and be identified. Therefore, we wish to explore how we can visualise this data and ask: how can we clearly represent the flow of annotation through the database?

A number of approaches to visualising large datasets were considered. One such approach is to model the relationship between sentences and entries as a graph. Using a tool, such as Cytoscape [22], we can easily model sentences occurring within entries (and vice versa). However, our experience with this approach suggests that it is troublesome to model change over time and manual intervention is often required to ensure nodes are organised in a correct and meaningful manner. Other similar approaches, such as Sankey diagrams, were not utilised as we cannot determine the exact source and flow of an annotation between each individual entry.

One approach which produces a visualisation similar to our requirements is the history flow tool [23]. This tool was developed to allow visualisation of relationships between multiple versions of a wiki. Therefore, it aims to clearly depict the change in sentences, and their order, in a document over time with the ability to attribute each change to a given author. The authors demonstrated this visualisation with an exploratory analysis of Wikipedia, revealing complex patterns of cooperation and conflict between Wikipedia authors. However, using the history flow tool to visualise the flow of individual sentences in UniProtKB is not ideal; crucially, the tool cannot clearly represent the data due to the disjoint nature of early Swiss-Prot and TrEMBL releases.

**Figure 9:**
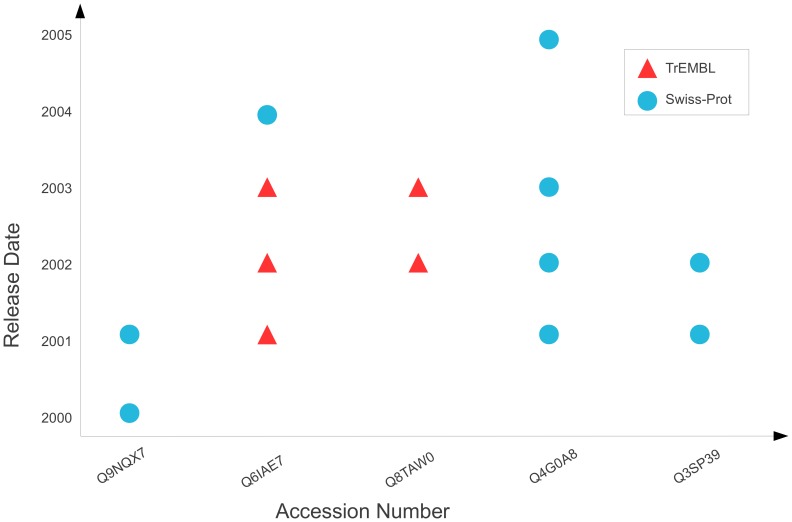
Manual illustration showing how the propagation of a single sentence could be visualised. Accession numbers are shown on the X-Axis, with database release dates shown on the Y-Axis. A point on the graph represents that the sentence occurs in an entry within a given database version. For example, the bottom left point shows that the sentence occurs in accession entry Q9NQX7 for Swiss-Prot in 2000 – this sentence remains in Q9NQX7 for one more version; it is removed in the following version (in 2002).

Given these issues, we look at creating a visualisation view of the annotation space that overcomes these restrictions, whilst also aiming to make the visualisation as intuitive as possible. We outline a visualisation approach, as manually illustrated in Figure 9, that is somewhat similar to a regular scattergraph plot and draws upon the strengths from the history flow tool. This approach allows propagation to be visualised whilst also remaining intuitive; we show each accession the sentence occurs within along the X-axis, with the Y-Axis showing the release date for the corresponding database versions. Therefore, a point on this graph represents the sentence occurring within an accession for a given database version, where a red point represents the sentence being in a TrEMBL entry, and a blue point represents a Swiss-Prot entry.

This approach quickly becomes cumbersome as the amount of data increases, specifically making it difficult to explore and examine individual data points. Additionally, there can be several TrEMBL releases for each Swiss-Prot release. This makes it appear that the sentence is constantly being removed and re-added; i.e. it exhibits *striping*. This striping is due to early releases of TrEMBL and Swiss-Prot being unsynchronised. One possibility to overcome the issue of striping is binning. However, this would lose a major level of granularity, as we would have to bin for every six months to cover all Swiss-Prot releases.

**Figure 10:**
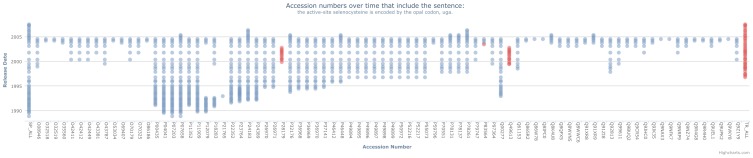
Visualising sentence propagation. Visualising the propagation of the sentence “the active-site selenocysteine is encoded by the opal codon, uga.” through the database, with all possible versions of Swiss-Prot and TrEMBL within this range shown at either end of the graph.

**Figure 11:**
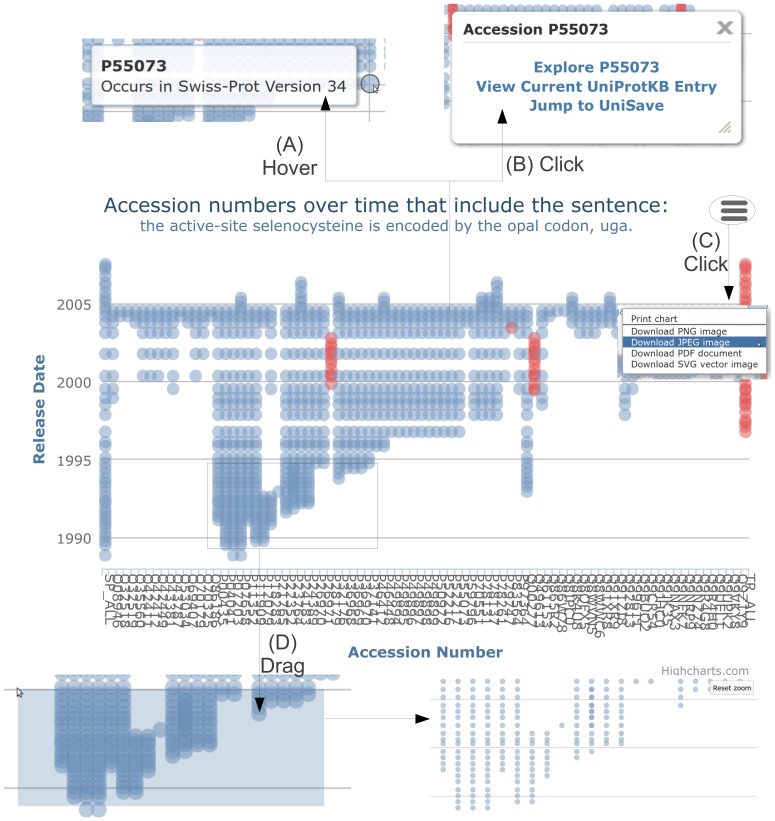
Illustrating the key interactive features provided by Highchart graphs. (A) Hovering over a point indicates the corresponding accession number and database version. (B) Clicking on a point provides links to the UniProt entry and further information. (C) Each graph can be printed and exported into a variety of image formats. (D) The ability to zoom into a section of a graph; this can be achieved by left-clicking and dragging a desired area.

To overcome these issues, we explored generating graphs using an interactive framework. The resulting graph, for the sentence “the active-site selenocysteine is encoded by the opal codon, uga.” is show in Figure 10. These graphs make use of Highcharts, an interactive charting library in JavaScript (http://www.highcharts.com/). This approach provides an interactive web-based chart option, that can be easily generated for any sentence. Further, we have the ability to zoom into dense graphs, hover over a point to clearly see the entry and corresponding database version and export graphs (i.e. save to file). These features, as illustrated in Figure 11, allow us to overcome the issues caused by dense graphs. Additionally, we show the release points for Swiss-Prot down the left side and TrEMBL down the right, thus making it clearer when a point is missing and further alleviating the striping issues.

Within the UniProtKB database, it is relatively common for entries to become merged. When a merge occurs, the entry has a single primary accession, with the merged entries becoming secondary accessions. Within our graphs, we show an entry by all of its accessions; not doing so would be misleading, as it would appear that an annotation has been removed when, in reality, the entry has been merged.

The development of this visualisation approach allows us to investigate further how individual sentences have been used within UniProtKB, and how they move between different entries over time. We next discuss how we have used this visualisation strategy.

### Exploring the annotation space: Can provenance be identified?

We have shown that sentence reuse is frequent within UniProtKB and is increasing as UniProtKB matures. With the development of a visualisation technique, we are now able to visualise sentence propagation, with the specific aim of investigating the provenance of annotations.

Taking as an initial example the sentence “the active-site selenocysteine is encoded by the opal codon, uga.”, we show the visualisation of its propagation in Figure 10. This graph shows that the sentence initially occurs in two entries in Swiss-Prot Version 9; P07658 and P07203 (the leftmost point, SP_ALL, is for illustration and used to alleviate striping, as previously discussed). In this particular instance, the provenance is between two entries – we cannot trace this further back as Swiss-Prot versions 1–8 and 10 are missing. Additionally, our level of granularity shown within these graphs is at major release level. Therefore, it is possible that a sentence will appear to originate in two or more entries within a single database version, when a distinction between them could be made at the minor release level. Minor release data was not parsed as it is only accessible through UniSave.

**Figure 12:**
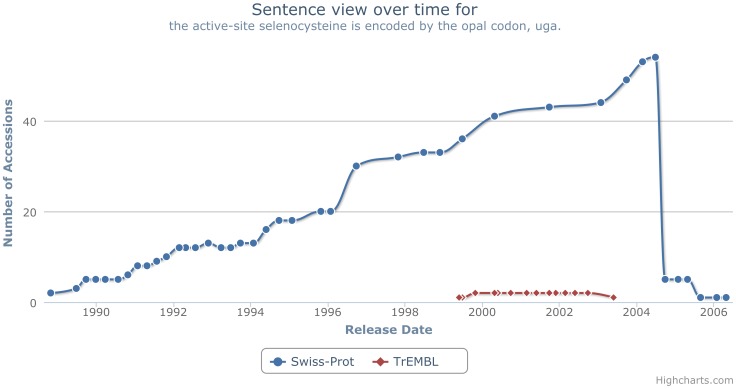
Visualising sentence occurrences. The number of UniProtKB entries that the sentence “the active-site selenocysteine is encoded by the opal codon, uga.” appears in over time.

Having identified that the sentence originated in two entries, we can also show that, at its peak, it was most commonly seen in Swiss-Prot Version 44, where it was found in a total of 54 Swiss-Prot entries, as illustrated in Figure 12. In total, the sentence appeared in 84 unique entries within UniProtKB until its removal.

The removal of this sentence was due to formatting changes within UniProtKB. Essentially, a change in the UniProtKB protocol meant that information about selenocysteine encoding moved from the textual annotation to the feature table of UniProtKB entries. Such technical changes are inevitable given the age of UniProtKB, which is constantly evolving to meet the requirements of new and updated developments. Such technical changes will inevitably impact a number of sentences. However, in this particular case we have identified a sentence that should have been replaced in all entries.

**Figure 13:**
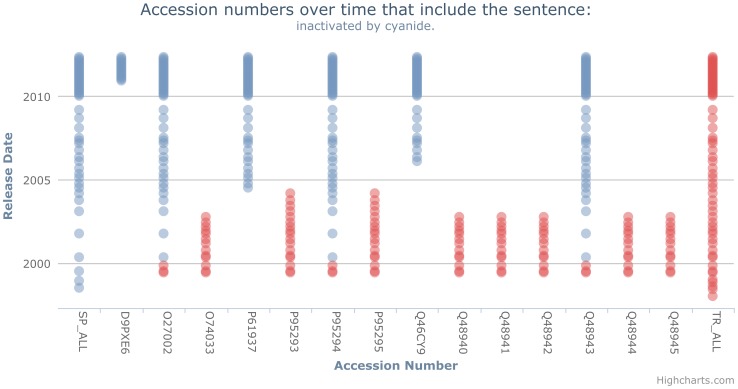
Visualising a sentence originating in TrEMBL. An example of a sentence (“inactivated by cyanide.”) that originates in TrEMBL, but ends up in Swiss-Prot. In this case, a number of the TrEMBL entries are merged into Swiss-Prot.

By analysing the flow of the sentence throughout UniProtKB in Figure 10, we notice a number of interesting *propagation patterns*:

- **Missing origin** – The sentence is removed from the entries it first originated in, yet still remains in a number of other entries in the database after this point.
- **Reappearing entry** – In two entries (P18283 and P12079) the sentence is removed, with the sentence actually being re-added to each of these entries after a number of versions have elapsed.
- **Transient appearance** – In a number of entries, such as P21765, the sentence only appears for a single version. It is removed from the subsequent release.
- **Originating in TrEMBL** – Although not shown in Figure 10, there are cases where a sentence originates in TrEMBL, before being propagated into Swiss-Prot entries. An example of this pattern is shown in Figure 13.

It is clear that by visualising sentences in this manner we are able to detect provenance. Moreover, inspection of these graphs has led to the discovery of a set of propagation patterns. These patterns are unexpected; why, for instance, should a sentence appear in only a single version of UniProtKB, or should a sentence disappear in an originating entry, but remain in an apparently derived entry? Given this, we now wish to examine these patterns further, exploring how frequently each pattern occurs within the database and what quality information can be drawn from them.

### Exploring the annotation space: Analysing propagation patterns

In the previous section, four propagation patterns were identified through the examination of sentences such as “the active-site selenocysteine is encoded by the opal codon, uga.”. If these patterns are of analytical value, then we would suspect a significant number of sentences adhering to each pattern to exist within the database. To obtain a list of these sentences, we used a series of set operations. For example, to identify sentences which follow the missing origin pattern, we take two sets: the entry, or entries, that the sentence first occurred within and the entries that the sentence last occurred within. If taking the intersection of these sets results in an empty set, then we have identified a sentence that is missing its root origin. Using this approach, an algorithm was created to allow the automated identification and extraction of sentences for each of the identified patterns.

Many sentences which exhibit each pattern were extracted from the UniProtKB database, with these results summarised in Table 1. In total, over 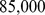 sentences follow at least one of the identified patterns, with over 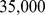 sentences remaining in the latest version of UniProtKB; in other words, approximately 9% of the unique sentences in UniProtKB Version 2012_05 follow one of the identified patterns. Amongst these patterns, transient sentences are the most prominent, accounting for approximately 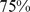 of the sentences following one of these patterns.

**Table 1:** Number of identified propagation patterns.

Pattern	Number of sentences	Number in just UniProtKB Version 2012_05
Missing Origin	8355	3835
Reappearing Entry	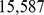	7011
Transient appearance	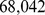	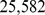
Originating in TrEMBL	8649	5330

The number of sentences that adhere to each pattern, for all versions of UniProtKB and those just in the latest version of UniProtKB. To place these results in context, there have been a total of 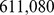 unique sentences, with 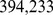 unique sentences being in UniProtKB Version 2012_05.

We have defined a transient sentence as one which is only present in an entry for a single database release before removal. By revisiting Figure 10, it can be seen that there are six instances for the given sentence where this occurs. Five of these cases occurred in Swiss-Prot version 44, when the majority of sentences were removed. The other case only occurs in entry P21765 for Swiss-Prot version 24, where the sentence is replaced by “the active-site is not encoded by the opal codon uga but by ugc.”. This replacement indicates that the knowledge in the original annotation is now considered erroneous. Our definition of erroneous annotation follows that of UniProt [24]: An erroneous annotation is one that is out of sync with respect to the biological knowledge. Indeed, it may be that the original information is incorrect, rather than the annotation.

Because of its nature, we can only detect transient sentences in the release before the current; a sentence must be added and then removed from the next release cycle. However, this pattern fits with previous research that links annotation quality to stability [25]; annotations that are persistent over many release cycles provide greater confidence and likelihood in their correctness. Therefore, using this information we can conclude that the introduction of an annotation within an entry update is more likely to be volatile than those which have remained over numerous releases. Importantly, transient sentences should not be seen as a burden to the overall quality of a database but used to indicate the importance of annotation maturity.

Although less common than transient sentences, over 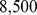 sentences in Swiss-Prot appear to originate from TrEMBL. This is a surprising observation; annotations in Swiss-Prot are considered manually reviewed and curated. Further, TrEMBL annotations can be generated based upon information from Swiss-Prot annotations [26]. Although automated annotations on the whole are typically of lesser quality than their manual counterparts [9], as part of their incorporation into Swiss-Prot, they will have undergone manual review. One possible explanation for this is that, for a period of approximately two years, some annotations in TrEMBL appear to have undergone manual annotation [27]. This was likely a result of a change in annotation policy, and it is interesting that we are able to identify such changes through this approach.

Clearly, a quality analysis between these sentences and those originating directly from Swiss-Prot would be of value. However, this analysis is not straightforward; no annotation quality metric that can analyse individual annotations is available. This result does, however, highlight that annotation provenance should be clearly documented and available to users, especially given that research has suggested that users often assume annotations are of a consistent quality [28].

**Figure 14:**
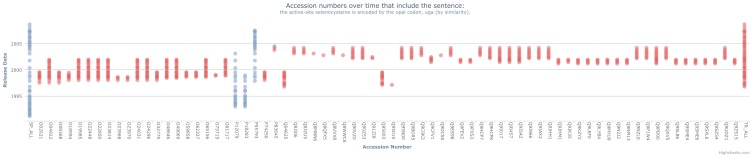
Visualising sentence propagation. Visualisation for the sentence “the active-site selenocysteine is encoded by the opal codon, uga (by similarity).”

Another interesting pattern observed is from those sentences which are removed from an entry, only to reappear in a subsequent release of the same entry; i.e. they follow the reappearing sentences pattern. In Figure 10 there are two examples of this pattern; the sentence reappears after 7 years in entry P18283 and 11 years in entry P12079. In these entries, the sentence was replaced with “the active-site selenocysteine is encoded by the opal codon, uga (by similarity).”, with the visualisation for this sentences shown in Figure 14. The usage of “by similarity” suggests that the information is based on sequence similarity. Interestingly, this sentence also follows the “missing origin” pattern.

Sentences exhibiting this pattern appear to indicate a conflict in the underlying evidence and some uncertainty as to the correct annotation. The impact of this pattern is similar to transient sentences; they highlight the importance of annotation stability and provenance.

The final pattern observed are sentences which are missing their root origins. Within Figure 10 the sentence initially occurs within two entries and remains in these entries until Swiss-Prot version 44, when it is subsequently removed. It is also removed from the majority of the other entries, however still remains in nine entries. Therefore, in Swiss-Prot Version 45 the sentence exists in these nine entries when it has been removed from the entries where it originated from. Depending on its reason for removal, it could highlight that possibly erroneous annotation still remains in the database.

However, as previously mentioned, this information was moved from the textual annotation to the feature table in UniProtKB entries. Therefore, this was not biologically erroneous in these nine entries. However, it clearly should have been moved to the feature table in all entries for consistency. This highlights how missing propagation of textual annotation can lead to inconsistencies between entries.

Changes in annotation are typically made to reflect an update in knowledge; in light of new knowledge a previous annotation may now be erroneous with respect to current knowledge. Given that annotations propagate, any updates to an original annotation should also be propagated. However, we identify over 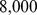 sentences which may, or may have, incorrectly remained in the database.

While these first three patterns are of interest in regard to annotation quality, we next investigate whether we can use the missing root origin pattern as an indication for erroneous annotations.

### Exploring the annotation space: Can we identify erroneous annotation?

As discussed in the previous section, over 8000 sentences exhibiting the missing origin pattern were identified. Here, we wish to analyse this pattern to support our hypothesis that it can be used to identify erroneous annotation. We define a missing origin sentence as one which:

1. Initially occurs in the *origin* entry.
2. Later appears in an additional entry; i.e. a *secondary* entry.
3. Is removed or changed in the origin entry.
4. Remains unchanged within the secondary entry for a subsequent database release (or releases).

Within this definition a sentence may also originate from, or propagate, to multiple entries. We determined that each sentence can be broadly categorised into one of five possible classifications:

- **Erroneous** The sentence in the secondary entry was inaccurate or incorrect given updates to the origin entry. This includes any case where detail is added or removed and not reflected in all relevant entries. This may include a sentence that has been reworded or one that has been removed entirely.
- **Inconsistent** Whilst the sentence in the origin entry has been updated, it has not changed the biological information contained within the sentence, or been propagated to the secondary entries. The correction of a grammatical error would be an example.
- **Accurate** The sentence in the secondary entry is accurate. Either the sentences appear identical by coincidence or the updates to the origin are not valid in the secondary entry. Therefore both annotations have become independent. For example, expression information may not be relevant in different organisms for the same gene.
- **Too many results** The sentence was very heavily reused within UniProtKB and deemed infeasible to analyse. The more entries that a sentence occurs within, the more troublesome it becomes to classify individually, given the vast number of entries they occur in. Specifically, sentences that occurs in over 100 are classified as “too many results”.
- **Possibly erroneous** Some sentences did not carry enough evidence, or contained conflicting information, making a more confident decision of classification impossible.

There are four main decisions when evaluating the classification of a textual annotation: deciding whether it is feasible to analyse the sentence; determining whether the sentence appears to have been copied between entries; deciding whether the update to the origin was relevant to the secondary entry and deciding whether the update affected the meaning of the textual annotation.

**Figure 15:**
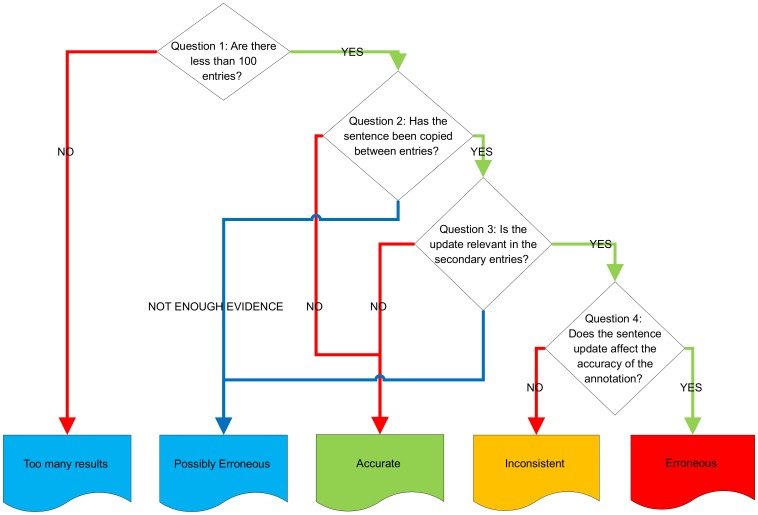
Decision tree summarising the protocol used to determine the classification of a sentence. There are four main questions within the protocol that lead to a sentence being classified into one of five possible classifications.

These decisions are subjective as interpretation of biological data can vary between users. Whilst our protocol attempts to allow for consistent interpretation, it is inevitable that reproducibility cannot always be attained between different users. Given this, a systematic and precise protocol was developed to encourage reproducible results. This protocol, represented as a decision tree in Figure 15, involves seven stages in determining a sentences classification:

1. Determine how many entries the sentence has propagated to. A sentence occurring in over 100 entries is infeasible to analyse (Figure 15, Question 1).
2. Using the visualisation framework, identify both the *origin* and *secondary* entries that the sentence occurs in.
3. Using the UniSave tool, analyse the context of the sentence within the origin and secondary entries at the time that the sentence was initially added to the secondary entry. Does this context suggest the sentence was propagated between the entries (Figure 15, Question 2)?
4. Determine the context for when the sentence was updated or deleted in the origin entry, then determine the context of the sentence in the secondary entry at the time when the sentence was deleted from the origin entry.
5. Is the update in the origin sentence relevant to the secondary entry (Figure 15, Question 3)?
6. Does the update in the origin entry affect the accuracy of the secondary entry (Figure 15, Question 4)?

**Figure 16:**
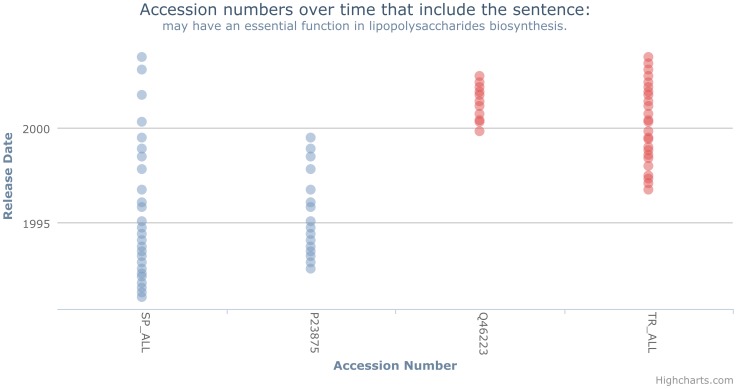
Visualising sentence propagation. Visualising the propagation of the sentence “may have an essential function in lipopolysaccharides biosynthesis.” through the database.

To illustrate this protocol, we can analyse the sentence “may have an essential function in lipopolysaccharides biosynthesis.”, for which the associated visualisation is shown in Figure 16. This sentence appears in less than 100 entries (step 1), with the sentence originating in a single entry (P23875) and being propagated to only a single secondary entry (Q46223) (step 2). Analysing the context of the origin (http://www.uniprot.org/uniprot/P23875.txt?version=11) and secondary entries (http://www.uniprot.org/uniprot/Q46223?version=7&version=6) at the time the sentence was added to the secondary entry shows significant overlap (step 3). For example, information relating to pathway information is also propagated. When the sentence is removed from the origin entry (http://www.uniprot.org/uniprot/P23875?version=11&version=12), the context appears relevant to the secondary entry (step 4). Given this, it appears that the removal of this information should also be applied to the secondary entry (step 5). Therefore, the sentence is classified as erroneous (step 6). Indeed, this sentence is eventually removed from the secondary entry (http://www.uniprot.org/uniprot/Q46223?version=12&version=11). When the sentence was removed from the origin entry (P23875) it was replaced with a cautionary topic stating that it was initially believed to have a function in lipopolysaccharides biosynthesis. This suggests that an update in knowledge meant the old annotation is now incorrect. When the sentence was removed from the secondary entry (Q46223), after ten database releases (three years), it was removed along with all other comments. Lipopolysaccharides biosynthesis was also removed from the keyword list; the only reference to lipopolysaccharides biosynthesis within the entry is in the title of a referenced article. This suggests that the sentence could have been removed ten database releases (three years) earlier.

Using this protocol, we analysed a total of 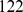 sentences; approximately 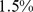 of the 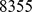 identified sentences. The complete set of analysed sentences are shown in Table 2, whilst the results are summarised in Table 3. A number of these 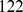 sentences were initially analysed to allow the protocol to be developed and refined. Additionally, a number of sentences under 20 characters long were analysed. To remove any sentence length bias a subset of sentences were taken; sentences were sorted by length and every hundredth sentence over 20 characters long was analysed. The decision to normalise for sentence length bias was based on the assumption that longer sentences are more likely to have greater information content. This resulted in 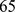 sentences being analysed, as summarised in Table 4.

**Table 2:** All analysed sentences and their classification.

Sentence	Classification
belongs to the 40s cdc5-associated complex (or cwf complex), a spliceosome sub-complex reminiscent of a late-stage spliceosome composed of the u2, u5 and u6 snrnas and at least brr2, cdc5, cwf2, cwf3, cwf4, cwf5, cwf6, cwf7, cwf8, cwf9, cwf10, cwf11, cwf12, cwf13, cwf14, cwf15, cwf16, cwf17, cwf18, cwf19, cwf20, cwf21, cwf22, cwf23, cwf24, cwf25, cwf26, cwf27, cwf28, ist3, lea1, msl1, prp5, prp10, prp12, prp17, prp22, sap61, sap62, sap114, sap145, slu7, smb1, smd1, smd3, smf1, smg1 and syf2.	Inconsistent
the light chain is composed of three structural domains: a large globular n-terminal domain which may be involved in binding to kinesin heavy chains, a central alpha-helical coiled-coil domain that mediates the light chain dimerization; and a small globular c-terminal which may play a role in regulating mechanochemical activity or attachment of kinesin to membrane-bound organelles (by similarity).	Erroneous
the biological conversion of cellulose to glucose generally requires three types of hydrolytic enzymes: 1) endoglucanases which cut internal beta-1,4-glucosidic bonds; 2) exocellobiohydrolases that cut the dissaccharide cellobiose from the nonreducing end of the cellulose polymer chain; 3) beta-1,4-glucosidases which hydrolyze the cellobiose and other short cello-oligosaccharides to glucose.	Inconsistent
in the hair cortex, hair keratin intermediate filaments are embedded in an interfilamentous matrix, consisting of hair keratin-associated protein (krtap), which are essential for the formation of a rigid and resistant hair shaft through their extensive disulfide bond cross-linking with abundant cysteine residues of hair keratins.	Inconsistent
the beta subunit of voltage-dependent calcium channels contributes to the function of the calcium channel by increasing peak calcium current, shifting the voltage dependencies of activation and inactivation, modulating g protein inhibition and controlling the alpha-1 subunit membrane targeting (by similarity).	Erroneous
interacts with the c-terminal of peptidylglycine alpha-amidating monooxygenase (pam) and may act as part of a signal transduction system linking the catalytic domains of pam in the lumen of the secretory pathway to cytosolic factors regulating the cytoskeleton and signal transduction pathways.	Erroneous
The modification is dependent on dna and is involved in the regulation of various important cellular processes such as differentiation, proliferation, and tumor transformation and also in the regulation of the molecular events involved in the recovery of cell from dna damage (by similarity).	Erroneous
adenosylhomocysteine is a competitive inhibitor of s-adenosyl-l-methinine-dependent methyl transferase reactions; therefore adenosylhomocysteinase may play a key role in the control of methylations via regulation of the intracellular concentration of adenosylhomocysteine (by similarity).	Inconsistent
component of the multisynthetase complex which is comprised of a bifunctional glutamyl-prolyl-trna synthetase, the monospecific isoleucyl, leucyl, glutaminyl, methionyl, lysyl, arginyl, and aspartyl-trna synthetases as well as three auxiliary proteins, p18, p48 and p43 (by similarity).	Erroneous
self; 2; ebi-311928, ebi-311928; p03949:abl-1; 4; ebi-311928, ebi-2315883; q17539:c01b10.8; 5; ebi-311928, ebi-311920; q95qi7:daf-3; 2; ebi-311928, ebi-326363; q09248:dnc-2; 2; ebi-311928, ebi-316282; q09975:lys-8; 2; ebi-311928, ebi-313861; q21831:snfc-5; 2; ebi-311928, ebi-360213;	Erroneous
the n-terminal of the protein extends into the stroma where it is involved with adhesion of granal membranes and photoregulated by reversible phosphorylation of its threonine residues; both are believed to mediate the distribution of excitation energy between photosystems i and ii.	Inconsistent
the modification is dependent on dna and is involved in the regulation of various important cellular processes such as differentiation, proliferation, and tumor transformation and also in the regulation of the molecular events involved in the recovery of cell from dna damage.	Erroneous
the iicd domains contain the sugar binding site and the transmembrane channel; the iia domain contains the primary phosphorylation site (the donor is phospho-hpr); iia transfers its phosphoryl group to the iib domain which finally transfers it to the sugar (by similarity).	Too Many Results
adenosylhomocysteine is a competitive inhibitor of s-adenosyl-l-methinine-dependent methyl transferase reactions; therefore adenosylhomocysteinase may play a key role in the control of methylations via regulation of the intracellular concentration of adenosylhomocysteine.	Inconsistent
this delta-9 desaturase is a terminal component of the liver microsomal stearyl-coa desaturase system, that utilizes o(2) and electrons from reduced cytochrome b(5) to catalyze the insertion of a double bond into a spectrum of fatty acyl-coa substrates (by similarity).	Inconsistent
in the absence of mercury merr represses transcription by binding tightly to the mer operator region; when mercury is present the dimeric complex binds a single ion and becomes a potent transcriptional activator, while remaining bound to the mer site (by similarity).	Erroneous
chemotactic-signal tranducers respond to changes in the concentration of attractants and repellents in the environment, transduce a signal from the outside to the inside of the cell, and facilitate sensory adaptation through the variation of the level of methylation.	Inconsistent
activated by tyrosine-phosphorylation in response to either integrin clustering induced by cell adhesion or antibody cross-linking, or via g-protein coupled receptor (gpcr) occupancy by ligands such as bombesin or lysophosphatidic acid, or via ldl receptor occupancy.	Erroneous
laminin is a complex glycoprotein, consisting of three different polypeptide chains (alpha, beta, gamma), which are bound to each other by disulfide bonds into a cross-shaped molecule comprising one long and three short arms with globules at each end (by similarity).	Erroneous
psi is a plastocyanin-ferredoxin oxidoreductase, converting photonic excitation into a charge separation, which transfers an electron from the donor p700 chlorophyll pair to the spectroscopically characterized acceptors a0, a1, fx, fa and fb in turn (by similarity).	Erroneous
involved in protection of chromosomal dna from damage under nutrient-limited and oxidative stress conditions.	Inconsistent
belongs to the cold-shock domain (csd) family.	Too Many Results
p35415:prm; 1; ebi-86215, ebi-133215;	Erroneous
composed of 14 different subunits.	Possibly Erroneous
proteins that associate with the core dimer include three families of regulatory subunits b (the r2/b/pr55/b55, r3/b″/pr72/pr130/pr59 and r5/b′/b56 families), the 48 kda variable regulatory subunit, viral proteins, and cell signaling molecules (by similarity).	Inconsistent
type i restriction and modification enzymes are complex, multifunctional systems which require atp, s-adenosyl methionine and mg(2+) as cofactors and, in addition to their endonucleolytic and methylase activities, are potent dna-dependent atpases (by similarity).	Inconsistent
3-beta-hydroxy-delta(5)-steroid + nad(+) = 3-oxo-delta(5)-steroid + nadh (acts on 3-beta-hydroxyandrost-5-en-17-one to form androst-4-ene-3,17-dione and on 3-beta-hydroxypregn -5-en-20-one to form progesterone).	Accurate
udp-n-acetyl-d-glucosamine + n-acetyl-beta-d-glucosaminyl-1,2-alpha-d-mannosyl-1,3(6)-(n-acetyl-beta-d-glucosaminyl-1,2-alpha-d-mannosyl,1,6(3))-beta-d-mannosyl-1,4-n-acetyl-beta-d-glucosaminyl-r = udp + n-acetyl-beta-d-glucosaminyl-1,2-(n-acetyl-beta-d-glucosaminyl-1,6)-1,2-alpha-d-mannosyl-1,3(6) -(n-acetyl-beta-d-glucosaminyl-1,2-alpha-d-mannosyl-1,6(3))-beta-d-mannosyl-1,4-n-acetyl-beta-d-glucosaminyl-r.	Erroneous
in e.coli rnase h participare in dna replication; it helps to specify the origin of genomic replication by suppressing initiation at origins other than the locus oric; along with the 5–′3éxonuclease of pol1, it removes rna primers from the okazaki fragments of lagging strand symthesis; and it defines the origin of replication for cole1-type plasmids by specific cleavage of an rna preprimer.	Inconsistent
thoracic aortic aneurysms and dissections are primarily associated with a characteristic histologic appearance known as m´  edial necrosisór érdheim cystic medial necrosisín which there is degeneration and fragmentation of elastic fibers, loss of smooth muscle cells, and an accumulation of basophilic ground substance.	Erroneous
component of the cleavage and polyadenylation specificity factor (cpsf) complex that play a key role in pre-mrna 3–′end formation, recognizing the aauaaa signal sequence and interacting with poly(a) polymerase and other factors to bring about cleavage and poly(a) addition (by similarity).	Inconsistent
there are two operons: the xylcab operon is responsible for the upper metabolic pathway from toluene to aromatic carboxylic acids, & the xyldlefg operon is required for the lower catabolic pathway from aromatic carboxylic acids to compounds that enter the trycarboxylic acid cycle.	Erroneous
hh is characterized by abnormal intestinal iron absorption and progressive increase of total body iron, which results in midlife in clinical complications including cirrhosis, cardiopathy, diabetes, endocrine dysfunctions, arthropathy, and susceptibility to liver cancer.	Inconsistent
prp is found in high quantity in the brain of humans and animals infected with the degenerative neurological diseases kuru, creutzfeldt-jacob disease (cjd), gerstmann-straussler syndrome (gss), scrapie, bovine spongiform encephalopathy (bse), etc. to other prp.	Accurate
involved in the atp-dependent selective degradation of cellular proteins, the maintenance of chromatin structure, the regulation of gene expression, the stress response, and ribosome biogenesis (by similarity).	Erroneous
coup (chicken ovalbumin upstream promoter) transcription factor binds to the ovalbumin promoter and, in cunjunction with another protein (s300-ii) stimulates initiation of transcription.	Inconsistent
the lys-124 ubiquitination also modulates the formation of double-strand breaks during meiosis and is a prerequisite for and dna-damage checkpoint activation (by similarity).	Erroneous
the export to cytoplasm depends on the interaction with a 14-3-3 chaperone protein and is due to its phosphorylation at ser-259 and ser-498 by camk (by similarity).	Erroneous
the sigma factor is an initiation factor that promotes attachment of the rna polymerase to specific initiation sites and then is released (by similarity).	Too Many Results
hydrolysis of 1,4-alpha-d-glucosidic linkages in polysaccharides so as to remove successive maltose units from the non-reducing ends of the chains.	Accurate
the resulting products may subsequently be converted to the corresponding alcohols that are incorporated into lignins (by similarity).	Erroneous
involved in the initial immune cell clustering during inflammatory response and may regulate chemotactic activity of chemokines.	Inconsistent
s-adenosyl-l-methionine + magnesium protoporphyrin = s-adenosyl-l-homocysteine + magnesium protoporphyrin monomethyl ester.	Erroneous
component of the coat surrounding the cytoplasmic face of coated vesicles located at the golgi complex (by similarity).	Accurate
hsp82 is an essential protein that is required by cells in higher concentrations for growth at higher temperatures.	Accurate
monoubiquitinated on lys-147; may give a specific tag for epigenetic transcriptional activation (by similarity).	Erroneous
probably a dodecamer composed of six biotin-containing alpha subunits and six beta subunits (by similarity).	Possibly Erroneous
organized into a structure (processome or rna degradosome) containing a number of rna-processing enzymes.	Inconsistent
involved in the formation of the nuclear envelope and of the transitional endoplasmic reticulum (ter).	Inconsistent
this methionine-rich region is probably important for copper tolerance in bacteria (by similarity).	Erroneous
they have identical ligand binding properties but different coupling properties with g proteins.	Possibly Erroneous
3-carboxy-2-hydroxy-4-methylpentanoate + nad(+) = 3-carboxy-4-methyl-2- oxopentanoate + nadh.	Accurate
this is a conceptual translation; two frameshifts had to be introduced to produce this orf.	Erroneous
component of the infraciliary lattice (icl) and the ciliary basal bodies (by similarity).	Possibly Erroneous
catalyzes the methylation of c-11 in precorrin-4 to form precorrin-5 (by similarity).	Possibly Erroneous
on the 2d-gel the determined pi of this unknown protein is: 6.2, its mw is: 28 kda.	Accurate
heterodimer of a p110 (catalytic) and a p85 (regulatory) subunit (by similarity).	Accurate
this viral protein may be involved in the regulation of the complement cascade.	Inconsistent
two forms; long (shown here) and short; are produced by alternative splicing.	Inconsistent
assembles at the inner surface of the cytoplasmic membrane (by similarity).	Too Many Results
1-aminocyclopropane-1-carboxylate + o2 = ethylene + hcn + co(2) + 2 h(2)o.	Accurate
bind preferentially single-stranded dna and unwind double stranded dna.	Inconsistent
involved in the regulation of hydrogenase expression (by similarity).	Erroneous
may have an essential function in lipopolysaccharides biosynthesis.	Erroneous
rch(2)nh(2) + h(2)o + acceptor = rcho + nh(3) + reduced acceptor.	Accurate
subunit 1 binds to the primer-template junction (by similarity).	Inconsistent
to immunoglobulin and major histocompatibility complex domain.	Too Many Results
isoform 3: membrane; multi-pass membrane protein (potential).	Possibly Erroneous
the beta subunit seems to be encoded by a multigene family.	Erroneous
atp + adenylylsulfate = adp + 3–′phosphoadenylylsulfate.	Inconsistent
an aryl sulfate + a phenol = a phenol + an aryl sulfate.	Erroneous
peptidyl-l-amino acid + h(2)o = peptide + l-amino acid.	Possibly Erroneous
in the c-terminus to yeast sla2 and c.elegans zk370.3.	Erroneous
mediates e2-dependent ubiquitination (by similarity).	Accurate
villin is a ca(2+)-regulated actin-binding protein.	Inconsistent
atp + undecaprenol = adp + undecaprenyl phosphate.	Accurate
aminoacyl-peptide + h(2)o = amino acid + peptide.	Inconsistent
to the calcitonin and to the secretin receptors.	Erroneous
heterodimer of an alpha chain and a beta chain.	Too Many Results
requires ca2+ and mn2+ ions for full activity.	Inconsistent
contains 1 immunoglobulin-like v-type domain.	Too Many Results
belongs to family 13 of glycosyl hydrolases.	Too Many Results
acts as a transglycosylase (by similarity).	Erroneous
nuclear effector molecule (by similarity).	Possibly Erroneous
involved in carbon catabolite repression.	Erroneous
q9vy42:cg1461; 1; ebi-194476, ebi-127720;	Erroneous
contains 6 ldl-receptor class b domains.	Erroneous
ring cleavage of 2,3-dihydroxybiphenyl.	Possibly Erroneous
not expected to have protease activity.	Accurate
secreted in hemolymph (by similarity).	Accurate
interacts with rad51 (by similarity).	Accurate
endplasmic reticulum membrane bound.	Accurate
associated with the plasma membrane.	Accurate
does not have a catalytic activity.	Possibly Erroneous
belongs to the eae/invasin family.	Erroneous
interacts with cyclin g in vitro.	Possibly Erroneous
self; 1; ebi-190958, ebi-190958;	Possibly Erroneous
binds 1 nickel ion per monomer.	Accurate
binds 1 magnesium per subunit.	Inconsistent
clavulanic acid biosynthesis.	Accurate
belongs to the ycf50 family.	Accurate
inhibited by acetazolamide.	Erroneous
involved in tumorigenesis.	Accurate
acetyltransferase enzyme.	Possibly Erroneous
phosphorylates ppp1r12a.	Possibly Erroneous
detected at low levels.	Accurate
interacts with trim28.	Accurate
contacts protein l19.	Erroneous
interacts with gcn5.	Accurate
may self-associate.	Accurate
secreted in milk.	Too Many Results
heme-thiolate.	Accurate
adipocytes.	Accurate
nadp.	Accurate
nuclear.	Too Many Results
p.	Too Many Results
25.	Too Many Results
1.	Too Many Results
3.	Too Many Results
2.	Too Many Results
venom.	Inconsistent
roots.	Inconsistent
leaf.	Inconsistent

All of sentences analysed, and their corresponding classification. Sentences have been stored in lowercase to allow for case insensitive comparison. For further information, including the entries affected by these sentences, please see the authors website.

**Table 3:** Sentence classification results.

Classification	Erroneous	Inconsistent	Accurate	Too many Results	Possibly Erroneous
Absolute	36	29	28	15	14
Percentage	29.5%	23.8%	23.0%	12.3%	11.5%
Potentially Erroneous	2465	1986	1918	1027	959

The classification results for all of the analysed sentences (122 in total).

**Table 4:** Classification of sentences over 20 characters in length.

Classification	Erroneous	Inconsistent	Accurate	Too many Results	Possibly Erroneous
Absolute	16	11	20	5	13
Percentage	24.6%	16.9%	30.8%	7.7%	20.0%
Potentially Erroneous	2057	1414	2571	643	1671

The classification results of the 65 sentences analysed, controlling for sentence length bias (i.e. every 100th sentence over 20 characters in length).

Our results show that 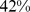 of sentences were identified as erroneous or inconsistent. Thirteen sentences were classified as “possibly erroneous”; in these instances we believed there was not enough evidence to confidently make a reasoned decision. This mostly arose when trying to determine sentence context (Figure 15, question 2). A similar number of sentences were classified as “inconsistent”, which suggests the curation process is asynchronized. The changes to these sentences were typically grammatical, often corrected after a number of versions. These issues could be overcome, or substantially reduced, if formal provenance were available.

Of these 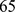 analysed sentences, 32 sentences remain in the most recent UniProtKB database release, with this subset of results summarised in Table 5. A breakdown of these results is provided on the supporting website.

**Table 5:** Classification of sentences in UniProtKB 2012_05.

Classification	Erroneous	Inconsistent	Accurate	Too many Results	Possibly Erroneous
Absolute	4	5	12	1	10
Percentage	12.5%	15.6%	37.5%	3.1%	31.3%
Potentially Erroneous	479	599	1438	120	1198

The classification of results for the subset of sentences controlling for sentence length bias analysed that remain in UniProtKB Version 2012_05.

Following up from these findings, we contacted the UniProtKB helpdesk to query our results. We submitted a detailed breakdown of three sentences to see if they agreed with our classification. For two of the three sentences, which are historical, they confirmed that if the sentence was to be re-added to the entry it would now be considered incorrect. For the final sentence, in the extant database, the origin sentence has been modified, and the current biological knowledge is not rich enough to determine whether the secondary entry is accurate; however, we suggest, our analysis raises a sensible question, that should be addressed as knowledge increases.

## Discussion

Current methods for detecting textual annotation provenance and tracking its propagation are somewhat limited. Within this paper we have presented a technique that allows annotation provenance and propagation to be identified and visualised by using sentences. Further, we have provided an analysis of sentence reuse levels and identified a number of annotation patterns that provide an indication as to an annotations quality and correctness.

The cornerstone of this work was dependent upon sentence reuse in UniProtKB. Our analysis shows that reuse is heavily prevalent for both Swiss-Prot and TrEMBL. This is because of the curation process employed by UniProt [6], which consists of six key stages, one of which involves identifying similar entries and standardising annotations between these entries. If two entries from the same gene and species are identified then they are merged. Therefore, sentences are effectively copied between entries as a matter of protocol. This process can see sections, or sometimes whole annotations, from one entry being copied to other entries without change.

Our analysis shows that this curation approach has decreased both the percentage of entries without annotation and increased the average number of sentences per entry over time. Whilst this appears to indicate an improvement in annotation coverage, in line with their stated goals, the actual annotation corpus is becoming more duplicated. We have shown that the average number of entries each sentence appears in is increasing, with the percentage of unique sentences in the latest version of UniProtKB being 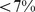 for Swiss-Prot and 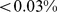 for TrEMBL. Similar patterns of reuse are also shown in work by Schnoes *et al.*, who have shown that the annotation of many high-throughput experiments is based upon a very small amount of experimental data [29].

Whilst the levels of reuse are generally increasing overtime, we interestingly note a slight decline in sentence reuse for later versions of Swiss-Prot. Although this decline coincides with the change of the UniProtKB release cycle, it appears to be related to a change in annotation policy for Swiss-Prot. After 2010 only sequences with experimental annotation were added to Swiss-Prot; previously automatically annotated orthologue sequences from complete genomes were often included in Swiss-Prot.

In the face of ever increasing raw biological data, this reuse is not unexpected. Whilst manual curation is often regarded as the ‘gold standard’ [30], it is a significant bottleneck. For example, in the FlyBase database it can take between two and four months for an article to be manually curated with consideration recently being given to incorporating sections of automated processing into the curation process [31]. It was for this same reason that UniProtKB introduced TrEMBL in 1996. Whilst reuse is understandably higher within automated methods, it is inevitably going to remain commonplace throughout both automated and manual databases while the quantity of raw biological data being generated continues to increase. Indeed, sentence reuse is an important feature of annotation curation. In addition to the propagation of knowledge, it also allows annotations to become standardised and can be used to enforce levels of quality control.

Whilst these results further highlight the importance of being able to identify the origin of an annotation, the analysis was only achievable given that UniProtKB make available all major historical versions of Swiss-Prot and TrEMBL. Users are typically only interested in the most recent and up-to-date biological data available, but this work highlights the added value and importance of being able to scour archival data; database features such as UniSave should be a requirement rather than a luxury.

It was this archival data that allows provenance and propagation to be analysed, allowing the development of a visualisation technique. These visualisations appear to be useful, as their usage allowed a number of propagation patterns to be identified.

Provenance is inferred by identifying the first UniProtKB entry that a sentence appears in. Similarly, the propagation of a sentence is viewed by determining all subsequent UniProtKB entries the sentence appears in over time. For individual sentences, this inference is not necessarily correct. For example, a sentence may originate in an entry outside of UniProtKB or within a minor release. Further, the appearance of a sentence in multiple entries may be an independent event, with no relationship between the entries. However, the curation process and levels of reuse identified would argue against this often being the case. More formal tracking of provenance within the database curation process would help to alleviate this difficulty.

Confidence that the apparent propagation of a sentence is correct can be gained by analysing the context that the sentences appear in, for example, by comparing sequence similarity. Indeed, performing such an analysis on the sentence “the active-site selenocysteine is encoded by the opal codon, uga.” led to the identification of four propagation patterns.

We believe that these identified patterns hold promise as quality and correctness indicators. For example, a sentence which adheres to the “reappearing entry” pattern could be considered more dubious, as its inclusion (or exclusion) within an entry is not definitive. Further, although not shown by the visualisations, a number of entries sharing a sentence have later become merged. For example, in the latest version of UniProtKB, accessions P22352; O43787; Q86W78; Q9NZ74 and Q9UEL1 are all merged into a single entry, with a sentence common to all entries remaining in the merged entry. Our analysis identified a number of sentences that adhere to each of the four patterns. These patterns were identified by manual inspection during the development of the visualisation framework. Further work could be undertaken to perform a comprehensive search to identify any additional propagation patterns. This work could also be extended to derive a quantitative metric. By combining these results with other textual metrics, such as Inverse Document Frequency (IDF) [32], annotations between entries could be scored and rated.

Deriving a quality metric is not straightforward. However, we hypothesised that the “missing origin” pattern could be used to identify erroneous annotations. This analysis is more discrete than deriving a quality measure, as a sentence can be classified into one of five groups. Our analysis identified a number of annotations we believe to be erroneous, including a number of sentences that still remain in the latest version of UniProtKB. As acknowledged earlier, these results are somewhat subjective. However, the UniProt help desk have checked our conclusions for three cases; in two cases these were correct, and in the third, we lack the biological knowledge to draw a definitive conclusion.

These results suggests that the identification of propagation patterns could aid in the discovery of erroneous annotations, and act as a mechanism to increase confidence into an annotations quality.

Within this paper we only analysed sentence propagation between major UniProtKB versions. UniProtKB versions prior to Version 2010_01 made the distinction between minor and major releases. Minor versions are not archived on the UniProtKB FTP server, but can be viewed interactively via the UniSave tool. A finer level of granularity could be achieved by extracting this information from UniSave and incorporating into our tool. This could unearth additional sentences that fit propagation patterns and may help distinguish the provenance of a sentence that appears to originate in two or more entries. In practice, however, this would be complex as the version numbers of UniSave and UniProtKB differ; exacerbating the problems caused by the lack of coordination between Swiss-Prot and TrEMBL releases.

This work could also be extended to analyse the evolution of individual sentences. Whilst sentences can be copied verbatim between entries, many sentences will be copied and then undergo minimalistic changes, such as the change of a single letter or word. By employing semantic similarity [33] coupled with a combination of historical data and IDF it may be possible to identify and track sentence, and annotation, evolution.

The structure and features of UniProtKB made it an ideal resource to perform this analysis. A clear extension to this work would be to apply the techniques and tools within this paper to other databases, allowing propagation and provenance to be identified. As noted earlier, it is plausible that annotations propagate *between* databases. For example, the InterPro database is used in the production of TrEMBL [34], whilst the neXtProt database integrates the annotation in UniProtKB/Swiss-Prot as a primary source, as well as incorporating data from a number of other sources such as GO and Ensembl [35]. With over 1500 active biological databases [36], if cross-database propagation does indeed occur, then the provenance map could be vast, and using this approach it is plausible that the “true” provenance and propagation of an annotation could be identified, and used to increase the quality of all databases. The visualisation tool was developed in a manner that will allow any textual resource to be compared.

Our initial analysis has provided a number of fruitful results. Extending this analysis to cross-database propagation and provenance could provide even more encouraging results and could take a significant step towards the ability to track and trace annotation propagation.
